# Why item response theory should be used for longitudinal questionnaire data analysis in medical research

**DOI:** 10.1186/s12874-015-0050-x

**Published:** 2015-07-30

**Authors:** Rosalie Gorter, Jean-Paul Fox, Jos W. R. Twisk

**Affiliations:** Department of Epidemiology & Biostatistics, VU university medical center, Amsterdam, Netherlands; EMGO+ institute for health and care research, Amsterdam, Netherlands; Department of Research Methodology, Measurement, and Data Analysis, Faculty of Behavioral, Management & Social Sciences, University of Twente, Enschede, Netherlands

**Keywords:** Longitudinal data, Hierarchical model, Item response theory, Questionnaires, Measurement error, Structural model, Plausible values, Multilevel model

## Abstract

**Background:**

Multi-item questionnaires are important instruments for monitoring health in epidemiological longitudinal studies. Mostly sum-scores are used as a summary measure for these multi-item questionnaires. The objective of this study was to show the negative impact of using sum-score based longitudinal data analysis instead of Item Response Theory (IRT)-based plausible values.

**Methods:**

In a simulation study (varying the number of items, sample size, and distribution of the outcomes) the parameter estimates resulting from both modeling techniques were compared to the true values. Next, the models were applied to an example dataset from the Amsterdam Growth and Health Longitudinal Study (AGHLS).

**Results:**

The results show that using sum-scores leads to overestimation of the within person (repeated measurement) variance and underestimation of the between person variance.

**Conclusions:**

We recommend using IRT-based plausible value techniques for analyzing repeatedly measured multi-item questionnaire data.

**Electronic supplementary material:**

The online version of this article (doi:10.1186/s12874-015-0050-x) contains supplementary material, which is available to authorized users.

## Background

In the field of medical epidemiological research, multi-item questionnaires are often used to measure the development of a subject’s health status over time. The resulting item observations are used as measurements of a continuous latent variable (i.e. a variable that is not directly observable). Examples of latent variables are health related quality of life [1, 2], and depression [3]. A measurement model is required to describe the relation between the observed categorical item responses (for example, Likert items with four answering categories: agree/slightly agree/slightly disagree/disagree) and the continuous latent variable.

To make statistical inferences about longitudinal measurements of the latent variable a statistical model is required that describes the development of the latent variable over time, while addressing the typical correlations between measurements of one person. The central question is how to measure the latent variable given the response data, and how to perform the longitudinal data analysis given the measured variables. In longitudinal designs, the data has a nested structure; i.e. repeated measurements are nested within the subjects. Due to the nested structure, the common independence assumptions between measurements do not hold and neither linear/logistic regression nor analysis of variance can be used in a straightforward way [4–8]. A multilevel model can be used to model the dependencies when there are multiple measurements nested within participants [9]. This multilevel modelling approach will be referred to as structural modeling to explore differences in longitudinal analyses with sum-scores and IRT-based scores as estimates for the latent variable.

Two fundamental theoretical frameworks can be used to measure latent variables given the response data. Historically, there is classical test theory (CTT), where sum-scores are the estimates of the latent variable. The other, theoretically more advanced framework is item response theory (IRT) where item response patterns are used to construct scores for the latent variable. Under CTT, item differences are ignored and sum-scores have a common measurement error variance across subjects. Under IRT, different scores are assigned to the different response patterns leading to the same sum-score, making it possible to distinguish between the latent variable scores of subjects with similar sum-scores. Item response patterns are lower-level observations and more informative about the latent variable than higher-level aggregated sum-scores, which ignore differences between response patterns leading to equal sum-scores. Another advantage of IRT is that the distribution of the latent variable can address skewness of the population distribution, where under CTT, the distribution of the latent variable is restricted to be symmetric. In most epidemiological studies however, a symmetric latent variable population distribution is not present [10, 11]. Despite the known benefits of IRT, epidemiological researchers are still using sum-scores [12–15] as estimators of the latent variable.

The measurement error associated with latent variables is usually ignored in the structural model when using sum-scores with equal amounts of measurement error for all scores on the latent variable. The parameter estimates of the structural model will be biased [16] consequently. To address the uncertainty associated with the measurements, the plausible value technology [17–21] can be used. In plausible value technology, several draws (mostly five [22]) from the posterior distributions of latent variable scores for each person are used as latent variables in the structural model. The results from the structural model are pooled for all draws to obtain parameter estimates. Plausible value technology can be used to address directly the negative implications of using sum-scores as measurements of latent variables, while making the comparison with IRT-based plausible values.

The objective of this paper is to stress the important differences between IRT and CTT for latent variable modeling in different situations and show why IRT measurement models should be used in longitudinal research. As a case study in epidemiological longitudinal data, the repeatedly measured trait anxiety questionnaire from the Amsterdam Growth and Health Longitudinal Study (AGHLS) [23] is used.

## Methods

### Structural model for longitudinal latent variables

A structural model (also known as latent regression model) describes the relationships between predictors and latent variables while addressing additional dependencies between the repeatedly measured latent variables. A well-known method to account for the nested structure of longitudinal data is multilevel modeling (or mixed modelling, random effects modelling, hierarchical linear modelling) as the structural model. Advantages of using multilevel modelling for longitudinal data analysis are that it is not necessary that subjects are measured on the same time points nor do follow up times need to be uniform. Furthermore, the model is capable of handling time-invariant and time-variant covariates. Also, it is possible to estimate subject-specific change across time. The following multilevel model will be considered,

1 $$ \begin{array}{c}{\theta}_{ij}={\beta}_j+{e}_{ij}\\ {}{\beta}_j=\gamma +{u}_j\end{array} $$

where *θ*_*ij*_ is the latent variable location of person *j* for measurement occasion *i*, *β*_*j*_ is the random intercept representing the average latent variable location of person j over measurement occasions; both error terms are normally distributed with *e*_*ij*_ ~ *N*(0, *σ*^2^), and *u*_*j*_ ~ *N*(0, *τ*^2^). The variance parameter *τ*^2^ of the multilevel model is the variance between persons (i.e. level-2 variance) whereas *σ*^2^ is the repeated measurement variance (i.e. the variance of the measurements within person; level-1 variance).

### Measurement part of the model

To estimate the latent variable *θ*_*ij*_ that is used in the structural model as described in equation Eq. 1, CTT or IRT-based methods can be used. Lord and Novick [24] pp. 44 describe the basic equation for the composition of the observed score, *X*_*gij*_, for the latent variable, *T*_*gij*_, for person *j* on measurement occasion *i* on questionnaire *g* as

2 $$ {X}_{gij}={T}_{gij}+{E}_{gij}, $$

where *T*_*gij*_ is the true score, and *E*_*gij*_ the error of measurement. The observed score consists of the true score and the error of measurement, which is assumed to be unbiased. When making this assumption about the measurement error, numbers can be attached to the answering categories of the items and summed over all items of the questionnaire. Then, a test score (i.e., sum-score) can be defined as

3 $$ {\theta}_{ij\_CTT}={\displaystyle \sum_{k=1}^K}{X}_{kij}, $$

where the response pattern for person *j* on measurement occasion *i* is given by (*X*_1*ij*_, …, *X*_*Kij*_), and where *K* represents the number of items in the questionnaire. These sum-scores are the CTT estimates for the latent variable and used as outcome variable in the longitudinal analysis (i.e. the structural model). There are two main problems with this way of quantifying latent variables. The first issue is that the characteristics of the test and the subject are inseparable, i.e. they cannot be interpreted without the other, which makes sum-scores population dependent. The second problem is that the standard error of measurement is assumed to be the same for all subjects, although some sum-scores are more informative about the latent variable than others. That is, it is much more likely that different subjects are measured with different precision. For example, extreme high or low sum-scores are more unreliable compared to average sums scores, meaning that the extreme sum-scores are less likely to distinguish between the subjects than the sum-scores in the middle of the scale.

The item response patterns are more informative about the latent variable than the aggregated sum-scores, which ignore differences between response patterns leading to the same sum-score. For example, when answering ‘yes’ to 10 out of 20 dichotomous items (1 = ' *yes* ', 0 = ' *no* '), the sum-score of 10 can be obtained in 20 !/10 ! ways. Under IRT different scores are assigned to the different response patterns all leading to the same sum-score, making it possible to distinguish the scores of respondents with similar sum-scores.

**Fig. 1:**
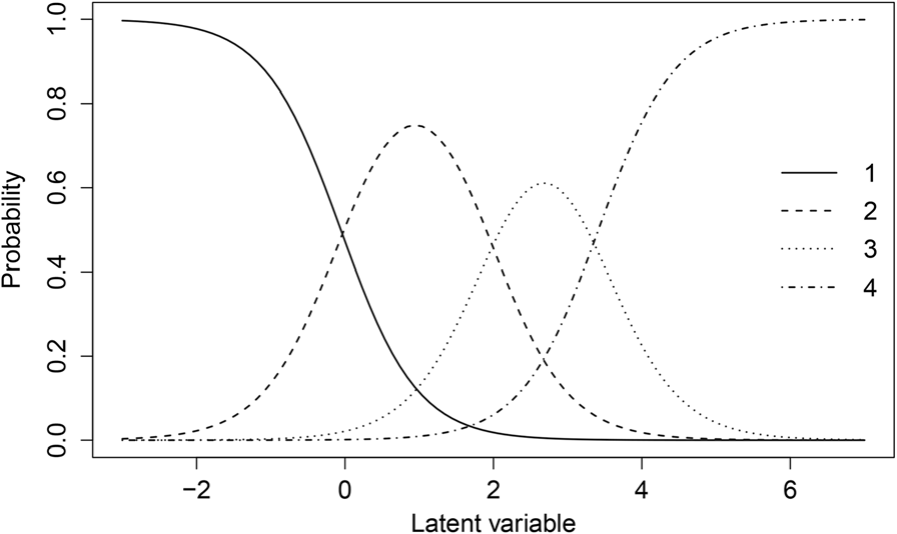
Item response characteristic curves for item 2 from the STAI-DY with four answering categories. The item that was used for this example was ‘I feel nervous and restless’ with four answering categories ‘1. Almost never’, ‘2. Sometimes’, ‘3. Often’, and ‘4. Almost always’. The crossing of two lines mark a threshold and can be interpreted as the location on the latent parameter where the probability of choosing the corresponding category or higher is 0.5

Using IRT modeling is an accepted way to account for the differences in measurement precision between persons [25–29] and can be used to estimate scores for the latent variable. In IRT, the relation between the unobserved latent variable *θ* and the observed item scores are described by item characteristic curves that model the probability of observed item responses. As a result, the item and latent variable estimates in IRT modeling are not dependent upon the population [30]. Fig. 1 depicts an example of item characteristic curves for an item with four response categories where the probabilities of choosing a certain category are plotted against the latent variable. An IRT model describes the relationship between latent variables and the answers of the persons on the items of the questionnaire measuring the latent variable [31]. For ordered response data, the probability that an individual indexed *ij* with an underlying latent variable *θ*_*ij*_, responds into category *c* (*c* = 1, . . . , *C*) on item *k* is represented by

4 $$ p\left({\boldsymbol{y}}_{ijk}=c\Big|{\theta}_{ij},{a}_k,{\tau}_k\right)=\phi \left({a}_k{\theta}_{ij}-{\tau}_{kc-1}\right)-\phi \left({a}_k{\theta}_{ij}-{\tau}_{kc}\right), $$

where *τ*_*kc*_ are the *C*_*k*_ − 1 threshold parameters. The probability that the response ***y***_*ijk*_ falls into category *c* is the difference of the probability densities (*ϕ*) of category *c* − 1 and category *c*. The response categories are ordered as − ∞ ≤ *τ*_*k*1_ ≤ *τ*_*k*2_ ≤ *τ*_*k*3_ ≤ ∞.

This item response model is called the graded response model [32] (or ordinal probit model [31, 33]). A Rasch [34] restriction was used fixing the discrimination parameters, *a*_*k*_, to one.

### Computing and generating IRT- and CTT-based scores

Different methods exist for generating values for the latent variable in the IRT framework. The first way is to generate point estimates of the latent variable modeled by the IRT model by constructing the posterior distribution of the latent variable given the data. An important assumption of IRT modeling is conditional independence, which entails that response probabilities for items rely solely on the latent variable, *θ*_*ij*_, and the item parameters. As a result, the joint probability of a response pattern ***y***_*ij*_, given the latent variable *θ*_*ij*_, of a person *j* on measurement occasion *i*, and given the item parameters, over the *K* items of the questionnaire is the product of the probabilities of the individual answers of a person on all items of the questionnaire given this person’s position on the latent variable *θ*_*ij*_ (equation Eq. 5).

5 $$ p\left({\boldsymbol{y}}_{ij}\Big|{\theta}_{ij}\right)=p\left({\boldsymbol{y}}_{ij1}\Big|{\theta}_{ij}\right)p\left({\boldsymbol{y}}_{ij2}\Big|{\theta}_{ij}\right)\dots p\left({\boldsymbol{y}}_{ijK}\Big|{\theta}_{ij}\right)={\displaystyle \prod_{k=1}^K}p\left({\boldsymbol{y}}_{ijk}\Big|{\theta}_{ij}\right), $$

When assuming a prior distribution for the latent variable distribution, *g*(*θ*_*ij*_), a posterior mean can be derived from the posterior *p*(*θ*_*ij*_|***y***_*ij*_) = *p*(***y***_*ij*_|*θ*_*ij*_)*g*(*θ*_*ij*_)/*p*(***y***_*ij*_), where the posterior is derived according to Bayes’ rule [35]. The posterior mean can be used as an estimate of the latent score.

When using the posterior mean as an estimate of the latent score and thus as an outcome variable in the structural model, the uncertainty associated with the score is ignored. To account for this uncertainty, plausible value technology is used [18, 19, 36, 37] where the latent outcome variable is treated as missing data. Plausible values are generated from the posterior distribution of the latent variable to obtain a complete data set. This data set can be analyzed in the secondary data analysis. When constructing the posterior of the latent variable, all available information is used. The posterior is proportional to the likelihood times the prior, which can be represented by

6 $$ p\left({\theta}_{ij}\Big|{\boldsymbol{y}}_{ij},{\sigma}^2,{\tau}^2,\boldsymbol{\gamma} \right)\propto p\left({\boldsymbol{y}}_{ij}\Big|{\theta}_{ij}\right)p\left({\theta}_{ij}\Big|{\sigma}^2,{\tau}^2,\boldsymbol{\gamma} \right). $$

The structural model parameters are integrated out such that the (marginal) posterior of the latent variable only depends on the response pattern. This marginal posterior is only dependent on the data, in this case upon the data of subject *j* on occasion *i*. We sample from the marginal distribution in order to obtain plausible scores for subjects with similar response patterns and background characteristics as in the sample of subjects [20, 21].

For the CTT model, the sum score defined in equation Eq. 3 is considered to be an unbiased estimate of the true score. This true score is considered to be an outcome of the multilevel model in equation Eq. 1. When assuming the CTT model for the measurement of the construct score, according to equation Eq. 2, the distribution of the observed scores given the true score is given by *p*(**y**_*ij*_|*θ*_*ij*_*CTT*_). Subsequently, the posterior distribution of the true score is given by

7 $$ p\left({\theta}_{ij\_CTT}\left|{\mathbf{y}}_{ij}\right.,{\sigma}^2,{\tau}^2,\gamma \right)\propto p\left({\mathbf{y}}_{ij}\left|{\theta}_{ij\_CTT}\right.\right)p\left({\theta}_{ij\_CTT}\left|{\sigma}^2,{\tau}^2,\gamma \right.\right). $$

Parallel measurements are needed to estimate the true score (error) variance, but they are usually not available. When the measurement error variance cannot be estimated under the CTT model, the first term on the right-hand side is not included in defining the posterior distribution, and an unbiased estimate of the true score is assumed. However, the measurement error can still be assumed to be included in the multilevel model specification (i.e., the second term on the right-hand side). In that case, the population variance is used as an approximation of the subject-specific measurement error variance [24] pp. 155. This approach was also used in the present study.

Analogue to generating plausible values under the IRT model (equation Eq. 6), the marginal distribution of the (true) scores is used to generate plausible values under the CTT model. Note that the drawn plausible values are realizations of the true score under the structural multilevel model given the sum score as an unbiased estimate of the true score.

In the literature, it is recommended to draw five sets of plausible values to address the uncertainty associated with the plausible values for the missing data [37, 38]. Various results of data analysis are obtained for the five different complete data sets, which are constructed from multiple sets of plausible values. The final results are constructed by averaging the analysis results, in this case, the structural model from equation Eq. 1.

### Comparing CTT and IRT-based estimates

When comparing the IRT and CTT-based structural model estimates, it is required to take scale differences into account. For the comparison, the CTT scores were rescaled to the IRT-based plausible values scale, using a linear transformation as proposed by Kolen and Brennan [39] pp. 337,

8 $$ sc(y)=\frac{\sigma (pv)}{\sigma (Y)}y+\left[\mu (pv)-\frac{\sigma (pv)}{\sigma (Y)}\mu (Y)\right], $$

where *μ*(*pv*) and *σ*(*pv*) are the mean and the standard deviation of the IRT-based plausible values, and where *μ*(*Y*), and *σ*(*Y*) are the mean and standard deviation of the CTT-based plausible values.

Next, the structural model was fit to the plausible values for each of the five draws. Finally, the estimates resulting from the structural model were pooled to obtain the final parameter estimates.

### Simulation study

A simulation study is presented for evaluating the use of IRT-based plausible values compared with CTT modeling for estimating latent variables used in longitudinal multilevel analysis. The aim of this study was to investigate how the true values of the population parameters are retrieved in different situations with varying sample sizes, number of items and skewness of the latent variable distribution.

### Design

**Fig. 2:**
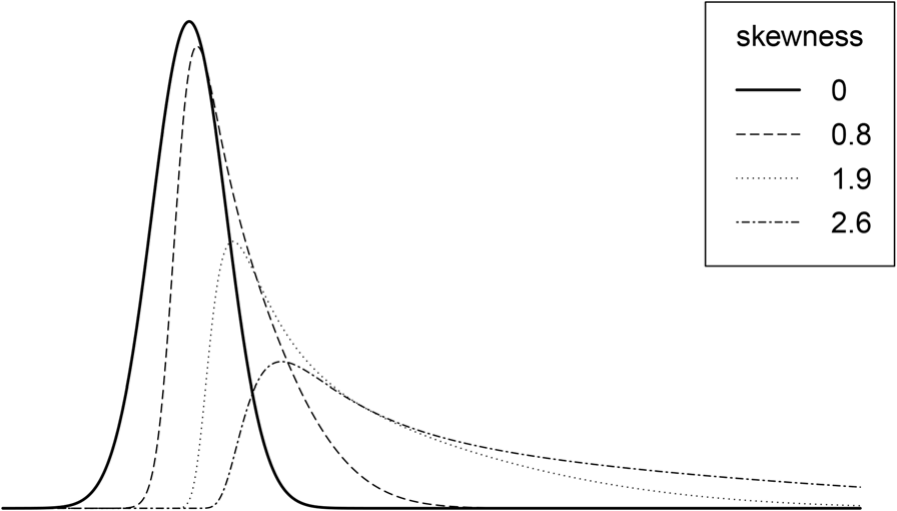
The density of four different skewed normal distributions for the latent variable

The full cross classified design resulting in 546 conditions is depicted in Table 1. Per condition, 10 datasets were simulated using R statistical software [40] and analyzed using an extended version of the R-Package mlirt [31] and WinBUGS [41, 42]. Data was generated following the model described in equation Eq. 1 with the variance between persons (i.e. level-2 variance) set to *τ*^2^=.8, and repeated measurement variance to *σ*^2^=.4 while using the IRT-model from equation Eq. 4 to generate values for the normally distributed underlying latent variable; *θ*_*ij*_ ~ *N*(0, 1). An unidimentional latent variable was assumed to cause the responses, measured six times *J* = 6 using a varying amount of items with four answering categories per item *C* = 4. The number of items that were used are listed in Table 1. In the simulated data, skewness of the latent variable was generated by changing the location of threshold parameters of the IRT model. For example, for a positive skewness of 2.6, *τ*_*k*1_ = 3, *τ*_*k*2_ = 2, and *τ*_*k*3_ = 1 were used for all items *k*. This skewed to the right data could indicate that relatively healthy persons were asked to answer a questionnaire measuring clinical depression, leading to high sum-scores. The same can occur when subjects have recovered after treatment and the same questionnaire is used on the baseline and follow up measurement. Item scores were generated based on the latent variable and the parameters of the IRT model. The CTT based scores are calculated according to equation Eq. 3 using the simulated item scores. These sum-scores are the CTT estimates for the latent variable. In Fig. 2, the distributions of the CTT scores are visualized using density plots of different skewness conditions. In epidemiological data, skewness in the data is often found. The influence of various levels of skewness of the latent variable distribution on retrieving the multilevel regression parameters was investigated.

**Table 1:** Simulation conditions. The conditions of the full cross classified design for the simulation study. The numbers of items, number of participants, as well as the skewness from a normal distribution of the latent variable were varied

Model^a^	Items^b^	N^c^	Skewness
IRT	3	100	0
CTT	5	500	+/− 0.4
	7	1000	+/− 0.8
	10		+/− 1.3
	15		+/− 1.9
	20		+/− 2.6
	50		+/− 3.6

^a^Measurement model that was used for drawing the plausible values for the latent variable

^b^Number of items

^c^Number of participants

**Fig. 3:**
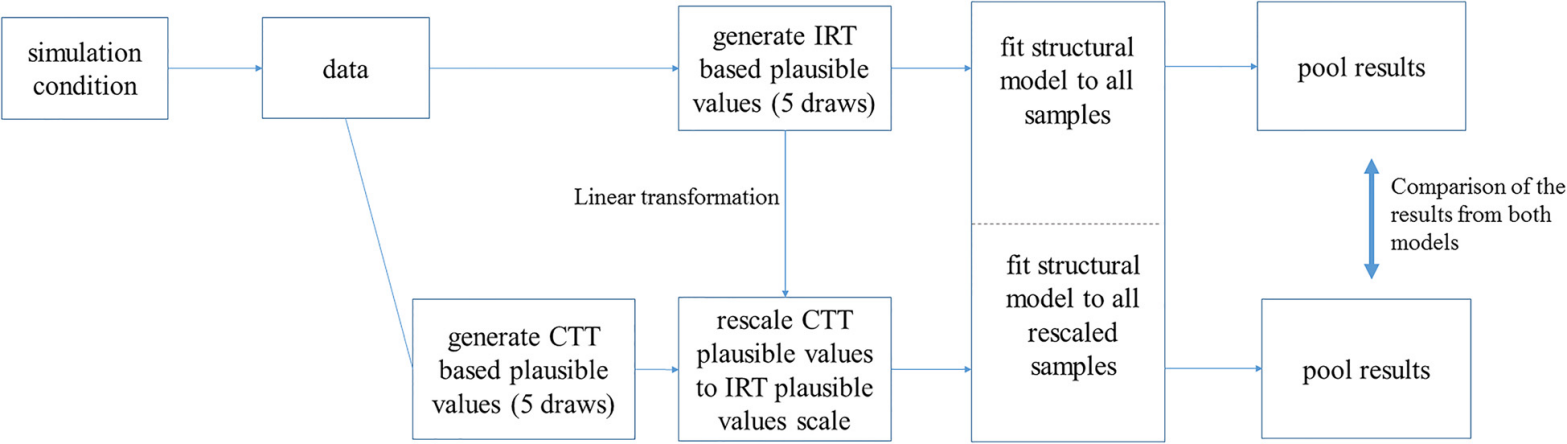
Simulation procedure for one replication

Figure 3 shows a schematic display of the simulation procedure for one replication. IRT and CTT-based plausible values were generated using the datasets from one of the simulation conditions. The CTT-based Plausible values were rescaled according to equation Eq. 7 and the structural model described in equation Eq. 1 was fit to five draws of plausible values and the results were pooled by averaging the parameter estimates. Mean squared errors (MSE) given by

9 $$ MSE\left(\widehat{\theta}\right)=Var\left(\widehat{\theta}\right)+{\left( Bias\left(\widehat{\theta},\theta \right)\right)}^2, $$

were calculated where $\widehat{\theta}$ denote the parameter estimates resulting from the different replications and *θ* are the true values.

The MSE’s were calculated for the level-1 and level-2 variance estimates for both the CTT and the IRT-based plausible value analysis.

### Simulation Results

**Fig. 4:**
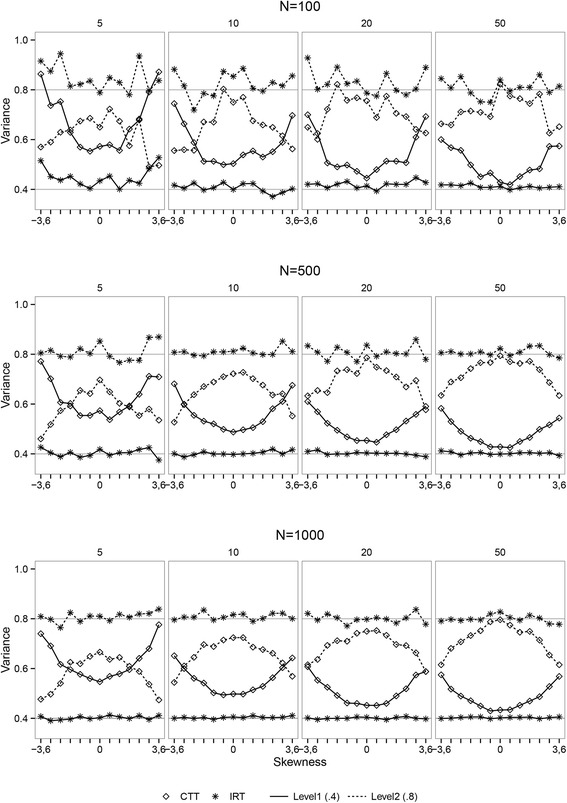
Pooled variance estimates. Selection of the pooled variance estimates for different distributions, sample sizes (*N* = 100, 500, 1000), and number of items (*K* = 5, 10, 20). The upper left plot for example represents 13 different skewness conditions (from negative to positive) with 100 participants measured on six time points with a five-item questionnaire. The points represent the IRT and CTT-based estimates for the level-1 (repeated measurement) and level-2 (between person) variance. The horizontal lines represent the true values for the variance parameters

**Fig. 5:**
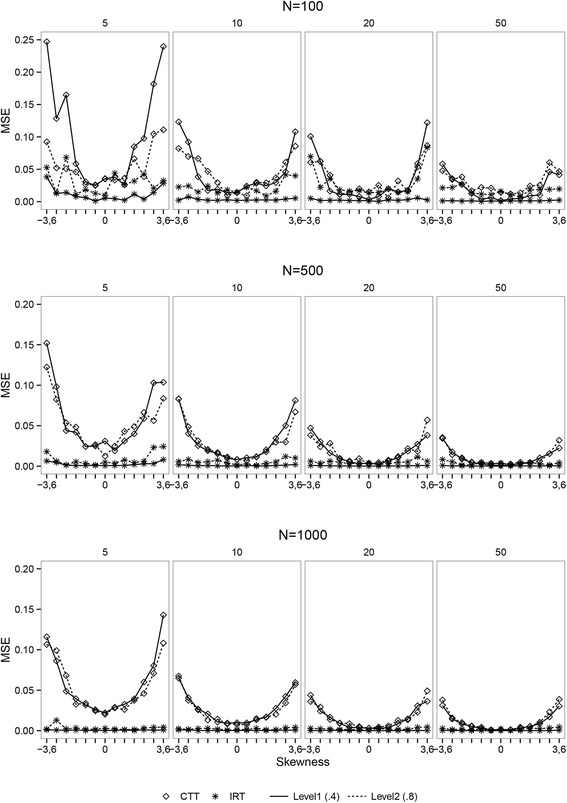
Mean squared errors. Plots of the MSE’s for level-1 (repeated measurement) and level-2 (between person) variance parameter estimates resulting from both the IRT and the CTT-based latent variable models

Figure 4 shows a selection of the variance estimates within persons (level-1) and between persons (level-2). The estimates for the IRT-based plausible value scores are closer to the true parameter value of 0.4 for the within person variance and 0.8 for the between person variance compared to the estimates from the CTT-based analysis. The difference between the methods is the smallest when the latent variable is perfectly normal distributed, and becomes gradually bigger with increasing skewness of the latent variable distribution. The estimates from the CTT model get closer to the true values when the number of items increase moving from the left to the right graphs. The estimates from the IRT model are close to the true values for number of item conditions with except for the *N* = 100 condition. The CTT repeated measurement variance estimates for the conditions with ten or less items are even higher compared to the between person variance estimates in case of more extreme skewness. This in contrary to the IRT-based estimates, where the variance estimates are very close to the true values regardless of the skewness. Overall, the IRT method gives more accurate estimates compared to the CTT model over all the simulation conditions. When comparing the plots from the top to bottom, the sample size increases from *N* = 100 to *N* = 500 to *N* = 1, 000. Increase in sample size does not influence the magnitude of the difference between both models. The IRT model gives better estimates in all sample size conditions. With the increasing sample size, the lines between the estimates become more stable, indicating a more stable pattern of the differences between both methods. The MSE’s for the variance estimates are presented in Fig. 5, where it can be seen that the CTT model systematically overestimates the repeated measurement variance and underestimated the between person variance. The differences between the true value and the estimated value by the CTT model increases when the latent variable distribution becomes more skewed. These differences become smaller for the CTT-based estimates when the number of items and the sample size increase. The observed difference between the IRT and CTT estimates seems to be dependent on the manipulated factors. The extremer data situations are causing larger differences between IRT and CTT-based estimates in a consistent way. A complete representation of the results can be found in Additional file 1 and Additional file 2 online.

### Empirical dataset

**Fig. 6:**
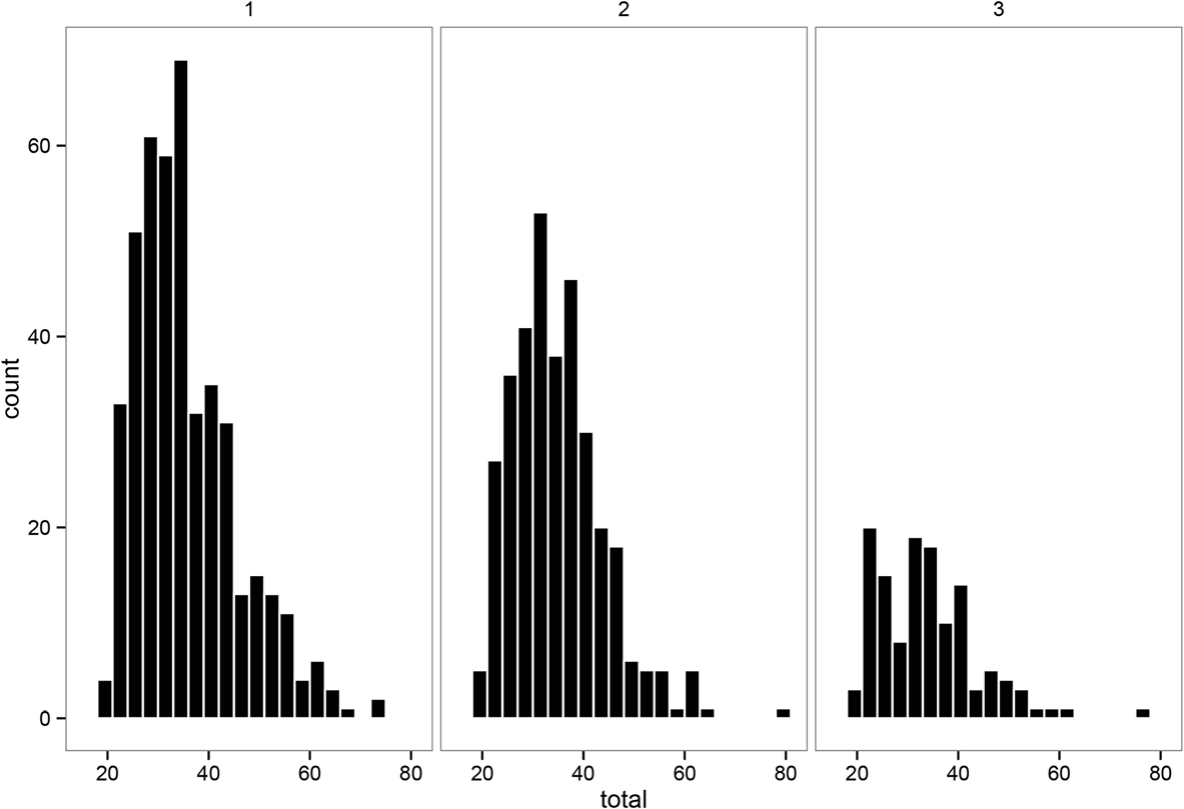
Sum-score distributions. Histograms with the sum-score distribution for the latent variable ‘state anxiety’ on the three measurement occasions in the AGHLS cohort. The skewness of the sum-score distributions was 1.02; 0.99; and 1.18 on the first, second, and third measurement occasion from left to right, and 443, 338, and 126 participants were included respectively

An example dataset was analyzed to illustrate the application of IRT-based plausible values in epidemiological practice. Data were obtained from the Amsterdam Growth and Health Longitudinal Study (AGHLS), which is a multidisciplinary longitudinal cohort study that was originally set up to examine growth and health among teenagers [43]. Data from the AGHLS were used in previous research to answer various research questions dealing with the relationships between anthropometry [44], physical activity [45], cardiovascular disease risk [46, 47], lifestyle [48, 49], musculoskeletal health, psychological health [50] and wellbeing. The presented sample consists of 443 participants who were followed over the period 1993–2006 with maximal three data points over time nested within the individuals. A subscale of the State Trait Anxiety Index Dutch Y-version (STAI-DY) [51] questionnaire was used to measure the latent variable ‘state anxiety’ and consists of 20 items with four answering categories. The histograms in Fig. 6 depict the sum-score distributions on the three measurement occasions. The aim of the analysis was to estimate the intercept and the variance parameters (i.e. an intercept only model) in order to compare the CTT and IRT-based estimates. The measurement models as well as the structural model that were used are comparable to those in the simulation study above.

## Results

**Fig. 7:**
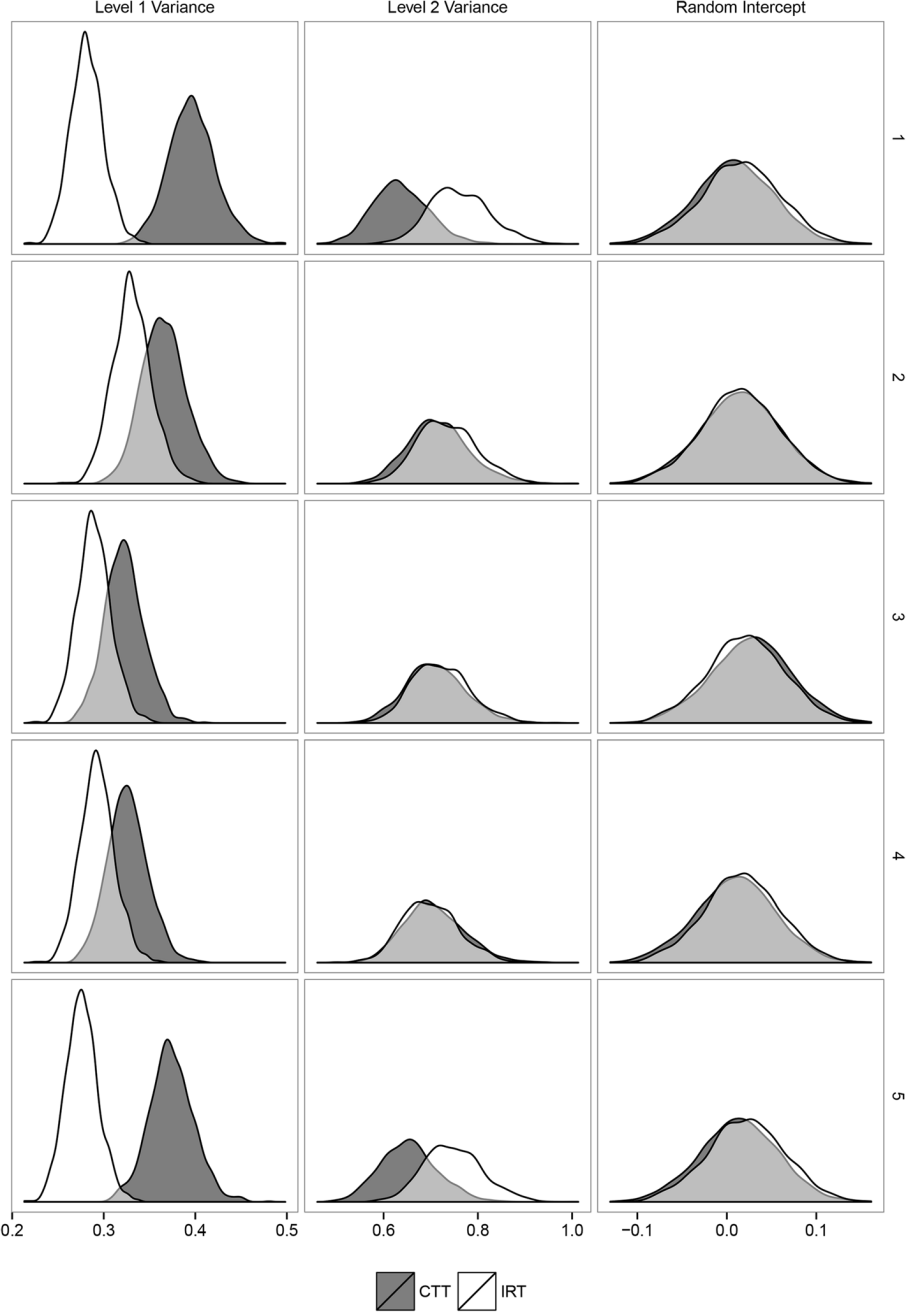
Posterior density plots. Posterior density plots for the level-1 (repeated measurement) variance, the level-2 (between person) variance, and the random intercept under the IRT and the CTT-based models for all five draws of plausible values

The pooled parameter estimates resulting from both the IRT and CTT-based models are presented in Table 2. The parameter estimates are derived by pooling the averages from the posterior distributions of the five draws of plausible values that are visualized in Fig. 7. Looking at the estimate for the random intercept on the first row of Table 2, it can be seen that the IRT and CTT-based estimates are similar. Furthermore, it can be seen that the between person variance is lower for the CTT-based model compared to estimates from the IRT-based model, 0.685 and 0.733 respectively. The within person variance was 0.357 for the CTT-based model while it was 0.294 for the IRT-based model. As a result, the intra class correlation coefficient (ICC) was higher for the CTT-based model indicating that there was relatively more residual variance relative to the total variance compared to the IRT-based model. As a result, the CTT-based model overestimates the ICC substantially. Looking at the posterior distributions resulting from the first draw of plausible values on the top row of plots in Fig. 7, the two distributions are overlapping partly however there is a clear difference between the locations of the IRT and CTT-based posterior density plots. In draw two, three, and four there is a difference in the level-1 (within person) variance posterior density, but no large difference for the level-2 (between person) variance posterior density. The posterior density plots for the random intercept estimates in all draws are overlapping almost completely, indicating no difference in the estimates of the random intercept between the IRT and CTT-based modeling techniques. The reason that the estimates for the intercept are the same for both models is the rescaling procedure that was used as described in equation Eq. 7. The mean and the variance for plausible values are rescaled to the same scale in order to guarantee the comparability of the estimates. The results from the data example are in concordance with the results from the simulation study, indicating that the IRT based estimates are closer to the true parameters.

**Table 2:** Results data example. Parameter estimates (posterior means) of the multilevel model for the example ‘Trait Anxiety’ data with a random intercept using the IRT-based plausible values technique and the CTT-based scores as outcome variables

	IRT^a^		CTT^b^	
	Mean^c^	SD	Mean^c^	SD
**Fixed effect**				
*γ* Intercept	0.018	0.043	0.015	0.045
**Random Effects**				
Between individual (level-2)				
*τ* ^2^ Intercept	0.733	0.067	0.685	0.074
Within individual (level-1)				
*σ* ^2^ Residual variance	0.294	0.030	0.357	0.042
**Intra Class Correlation**				
*ρ*	0.287		0.343	

^a^Item response theory based estimates

^b^Classical test theory based estimates using sum-scores

^c^Mean of the coefficients resulting from fitting the structural model (longitudinal multilevel model) to the five draws of plausible values based on the IRT or CTT measurement models

## Discussion

Despite the known benefits of IRT modeling when analyzing latent variables, CTT models are still used very often in the field of epidemiological research. The objective of this study was to point out the differences between the use of IRT-based plausible values and the use of sum-scores in the measurement part of a longitudinal analysis. In this study, it is shown that the common way of doing longitudinal analysis with sum-scores leads to systematically biased results and more advanced statistical methods are required to make profound inferences in longitudinal latent variable research. We showed that IRT-based plausible value techniques performs better compared with CTT analysis for retrieving variance estimates in longitudinal data with latent outcome variables measured with questionnaires. The difference between both methods becomes consistently larger for the more extreme conditions of the simulation, indicating that IRT-based plausible value techniques are quite robust against more extreme data situations. The bias in the CTT based estimates can be reduced by using a larger number of items, a larger sample size, and by using data following a strictly normal distribution. However, in almost all of the data situations in our simulation study, longitudinal IRT performs much better in retrieving the variance estimates. In practice, epidemiological questionnaire data is seldom normally distributed [52–54], and using IRT-based estimates can improve the quality of the estimates profoundly. The systematic underestimation of the between person variance and overestimation of the within person variance by the CTT-based model leads to overestimation of the ICC. This might have impact on the regression coefficients and cause bias. It would be interesting to investigate the sequence and direction of this bias and the impact on the conclusions of past and future research. Besides that, nowadays there is also much interest in using multilevel modeling to explain differences between individuals and groups, which makes it even more important to use unbiased estimates for the variance parameters. Based on the outcomes of our research, it is advisable to use IRT-based plausible value techniques when the outcome variable is a repeatedly measured latent variable, especially when the sum-score distribution deviates from strictly normal. Plausible values are not an estimator for the construct, they can never be used to make inferences about individuals. Like most statistical inferences, the objective is to make statements about or comparisons between groups of people.

In the work of Blanchin *et al.* [55], longitudinal data modeling results under classical test theory and Rasch IRT models have been compared. In their work, scale-free statistical results are compared as type-I errors and power, since the dependent (latent) variables are not measured on a comparable scale. The CTT and IRT-based analysis showed comparable results in terms of power. This is in contrast to our findings, where we showed a significant increase in bias under the CTT model. However, their comparison is more complex since a common test approach is used (i.e., *t*-test and F-test), which is based on different assumptions in the different modeling approaches. The accuracy of the approximation of the distribution of the test statistic is likely to vary over techniques and models, which could influence the statistical results. Furthermore, differences in estimation methods and modeling differences also influenced their results.

The current study was confined to latent variables measured using questionnaires with items containing four ordinal answering categories. Although this is a common situation, questionnaires with a dichotomous response format (i.e. two answering categories) are used as well for measuring latent variables. When using questionnaires with dichotomous response format there will be less variance in sum-scores compared to ordinal response formats. There are less possible response patterns leading to less variance in scores when aggregated into a sum-score. As a result, the difference in estimates between IRT and CTT will most likely become even larger in all situations.

The simulation study as presented, only took into account complete datasets. Further research is needed to explore the influence of missing data on the difference between both methods. Furthermore, the focus of the current article is the use of latent variables as outcomes in the structural model. Another interesting study would be to focus on the influence of using CTT based scores for time (in)variant covariates which is a common situation in epidemiological research [56].

## Conclusions

From this study it can be concluded that the use of IRT-based latent variable scores, in contrast to sum scores, leads to unbiased parameter estimates in longitudinal data analysis given multi-item questionnaire data. The degree of bias increases when the latent variable distribution is more skewed. It is important to realize that longitudinal data analysis results are biased when using sum scores.

## Additional files

Additional file 1:
**Variance estimates.** Contains a table with the IRT and CTT-based pooled variance estimates of the structural model resulting from all different condition of the simulation study. (XLSX 28 kb) Additional file 2:
**Mean squared errors.** Contains a table with the IRT and CTT-based mean squared errors for the variance estimates of the structural model resulting from all different condition of the simulation study. (XLSX 20 kb)

## Abbreviations

IRT: Item Response Theory
AGHLS: Amsterdam Growth and Health Longitudinal Study
CTT: Classical Test Theory
MSE: Mean Squared Error
STAI-DY: State Trait Anxiety Index Dutch Y-version
